# Evaluation of Statewide Restrictions on Flavored e-Cigarette Sales in the US From 2014 to 2020

**DOI:** 10.1001/jamanetworkopen.2021.47813

**Published:** 2022-02-10

**Authors:** Fatma Romeh M. Ali, Donna Vallone, Elizabeth L. Seaman, Jamie Cordova, Megan C. Diaz, Michael A. Tynan, Katrina F. Trivers, Brian A. King

**Affiliations:** 1 CDC Foundation, Atlanta, Georgia; 2 Truth Initiative, Washington, DC; 3 Office on Smoking and Health, National Center for Chronic Disease Prevention and Health Promotion, Centers for Disease Control and Prevention, Atlanta, Georgia

## Abstract

**Importance:**

e-Cigarettes are the most commonly used tobacco product among US youths. Flavors are among the most cited reasons for use of e-cigarettes among youths, and therefore, some states have imposed restrictions on flavored e-cigarette sales. To our knowledge, no study has compared e-cigarette sales between states with statewide flavored e-cigarette restrictions and states without such restrictions while controlling for co-occurring events.

**Objective:**

To assess whether implementation of statewide restrictions on flavored e-cigarette sales in Massachusetts, New York, Rhode Island, and Washington was associated with a reduction in total e-cigarette unit sales from 2014 to 2020.

**Design, Setting, and Participants:**

This cross-sectional study with difference-in-differences analysis used e-cigarette retail sales data from Massachusetts, Rhode Island, and Washington, which implemented restrictions on flavored e-cigarette sales in October 2019; New York, which implemented these restrictions in May 2020; and 35 states without these restrictions (control states). Sales were summed into 4-week periods from August 24, 2014, to December 27, 2020, for a total of 2988 state-period observations.

**Main Outcomes and Measures:**

A difference-in-differences analysis was conducted to compare e-cigarette unit sales in the 4 states with flavor restrictions (before and after implementation) with those in the 35 control states. The model controlled for other population-based policies and emergent events (eg, the COVID-19 pandemic). Data on 4-week e-cigarette unit sales were sorted into 4 flavor categories (tobacco, menthol, mint, and other). Unit sales were standardized to reflect the most common package sizes for each product type.

**Results:**

Statewide restrictions on non–tobacco-flavored e-cigarette sales were associated with the following reductions in mean 4-week total e-cigarette sales in intervention states compared with control states from October 2019 to December 2020: 30.65% (95% CI, 24.08%-36.66%) in New York, 31.26% (95% CI, 11.94%-46.34%) in Rhode Island, and 25.01% (95% CI, 18.43%-31.05%) in Washington. In Massachusetts, the comprehensive sales prohibition of all e-cigarette products was associated with a 94.38% (95% CI, 93.37%-95.23%) reduction in 4-week sales compared with control states. Except in Massachusetts, where all sales of flavored e-cigarettes decreased, reductions were found only for non–tobacco-flavored e-cigarette sales in the other states with restrictions. Among control states, mean sales decreased by 28.4% from August 2019 to February 2020 but then increased by 49.9% from February through December 2020.

**Conclusions and Relevance:**

In this cross-sectional study, statewide restrictions on the sale of flavored e-cigarettes in Massachusetts, New York, Rhode Island, and Washington were associated with a reduction in total e-cigarette sales. These findings suggest that not all e-cigarette users who purchased non–tobacco-flavored e-cigarettes switched to purchasing tobacco-flavored e-cigarettes after policy implementation.

## Key Points

**Question:**

Were statewide restrictions on flavored e-cigarette sales in Massachusetts, New York, Rhode Island, and Washington associated with a reduction in total e-cigarette unit sales from 2014 to 2020?

**Findings:**

In this cross-sectional study, a difference-in-differences analysis of e-cigarette retail data showed that statewide restrictions on non–tobacco-flavored e-cigarette sales were associated with reductions of 25.01% to 31.26% in total e-cigarette unit sales compared with total sales in states without restrictions. The reductions were attributable mostly to decreases in non–tobacco-flavored e-cigarette sales.

**Meaning:**

In this study, statewide restrictions on sales of non–tobacco-flavored e-cigarettes were associated with reductions in e-cigarette sales.

## Teaser

This cross-sectional study assesses the association of statewide restrictions on flavored e-cigarette sales in Massachusetts, New York, Rhode Island, and Washington with total e-cigarette unit sales from 2014 to 2020.

## Introduction

e-Cigarettes have been the most commonly used tobacco product among US youths since 2014.^1^ In 2021, current e-cigarette use was 11.3% (1.72 million) among high school students and 2.8% (320 000) among middle school students.^2^ Youth e-cigarette use is associated with multiple factors, including flavors.^3,4,5^ More than three-quarters of US youths and young adults who use e-cigarettes report they would no longer use e-cigarettes if they were not flavored.^6^

The 2009 Family Smoking Prevention and Tobacco Control Act^7^ prohibited the sale of cigarettes with any additional characterizing flavor other than menthol; the sale of other flavored tobacco products was not prohibited. In January 2020, the US Food and Drug Administration issued a policy prioritizing enforcement against sales of certain flavored cartridge-based e-cigarettes other than tobacco flavor or menthol^8^ and, in April 2021, announced its intention to prohibit the sale of menthol cigarettes and flavored cigars.^9^ However, states and communities maintain broad authority to adopt additional or more stringent requirements regarding tobacco product use, sales, marketing, and other topics; to date, at least 300 local jurisdictions have enacted policies to reduce the availability of flavored tobacco products, including menthol cigarettes.^10^ Some of these policies were short-term emergency actions in response to an outbreak of e-cigarette or vaping product use–associated lung injury (EVALI) in 2019, which was primarily linked to tetrahydrocannabinol-containing products from informal sources.^11^ During the EVALI outbreak in late September 2019,^10^ Massachusetts issued regulations that initially restricted the sale of all e-cigarettes in stores and online and were narrowed to restrict the sale of non–tobacco-flavored e-cigarettes as of December 2019.^12^ A new law took effect beginning June 1, 2020, that made these provisions permanent, prohibited menthol cigarette sales, and established a 75% excise tax on nicotine-containing e-cigarettes.^13^ New York prohibited non–tobacco-flavored nicotine e-cigarette sales as of May 17, 2020.^14^ In Rhode Island, an emergency order went into effect on October 4, 2019, that prohibited sale of all non–tobacco-flavored e-cigarettes and became permanent in March 2020.^12,15^ In Washington, an executive order prohibiting sale of non–tobacco-flavored e-cigarettes went into effect on October 10, 2019, and lasted for 120 days.^16^

Prior studies have evaluated flavored tobacco product restrictions at the municipal level in New York,^17,18^ Massachusetts,^19,20,21^ Minnesota,^22,23^ and California.^24^ However, to our knowledge, no study has compared e-cigarette sales between states with statewide flavored e-cigarette restrictions and states without such restrictions while controlling for co-occurring events. Therefore, this study used a difference-in-differences (DID) analysis to compare e-cigarette sales before and after implementation of restrictions in Massachusetts, New York, Rhode Island, and Washington with sales in control states that did not implement such restrictions. The model controlled for other population-based policies, emergent events (eg, EVALI outbreak and COVID-19 pandemic), state and time fixed effects, and state differences in sales trends and demographic and economic characteristics.

## Methods

### Data Source

This cross-sectional study with difference-in-differences analysis used e-cigarette retail sales data licensed from IRI^25^ that included universal product code weekly sales from a sample of convenience stores, gas stations, grocery stores, drug stores, mass merchandiser outlets, retail chain stores, club stores, dollar stores, and military bases; these data were estimated to cover 77% of these retail stores in the US. Sales from online retailers and vape shops were not available. Data from Alaska, Hawaii, and Montana were not commercially available. In addition, sales from Delaware, Idaho, Kansas, Minnesota, Mississippi, Nebraska, New Jersey, and New Mexico were excluded because their data did not include convenience stores, which represented 99.6% of national e-cigarette sales in 2020 based on IRI data.^25^ Sales were summed into 4-week periods from August 24, 2014, to December 27, 2020, for a total of 2988 state-period observations. The Advarra institutional review board determined that this research did not involve human participation and, therefore, did not require institutional review board oversight or informed consent. This study followed the Strengthening the Reporting of Observational Studies in Epidemiology (STROBE) reporting guideline.

### Measures

#### Intervention and Control States

Intervention states (those with statewide flavored e-cigarette sales restrictions) included Massachusetts, New York, Rhode Island, and Washington. Control states included 35 states with no statewide flavored e-cigarette sales restrictions: Alabama, Arizona, Arkansas, California, Colorado, Connecticut, Florida, Georgia, Illinois, Indiana, Iowa, Kentucky, Louisiana, Maine, Maryland, Michigan, Missouri, Nevada, New Hampshire, North Carolina, North Dakota, Ohio, Oklahoma, Oregon, Pennsylvania, South Carolina, South Dakota, Tennessee, Texas, Utah, Vermont, Virginia, West Virginia, Wisconsin, and Wyoming.

#### e-Cigarette Sales

To aggregate unit sales, units were standardized to reflect the most common package size for each product type to account for the variations in product type. Based on previous studies,^26,27^ a standardized unit was equal to 5 prefilled cartridges or pods, 1 disposable device, or 1 e-liquid bottle.

e-Cigarette flavors were categorized as tobacco, menthol, mint, or other (fruit; clove or spice; chocolate; alcoholic drink, such as wine, cognac, or other cocktails; candy, desserts, or other sweets; or another flavor) based on the flavor description in the data set.^27,28^ For example, products were classified as tobacco flavored if tobacco or a descriptor (eg, traditional, original) was mentioned in a flavor’s name. Ambiguous or concept flavors that could not be readily identified (eg, “fusion”), which represented 5.6% of sales, were searched online and categorized. Flavors that could not be classified accounted for less than 0.1% of sales. e-Cigarette accessories and devices sold without e-liquids were excluded (11.5% of sales). All sales data were obtained from IRI.^25^

#### Flavored e-Cigarette Restrictions

Dichotomous variables were created to indicate dates when flavored e-cigarette sales restrictions were in effect in intervention states. For Massachusetts, 2 dichotomous variables were created. The first indicator was equal to 1 from the 4-week period ending on November 3, 2019, to the 4-week period ending on December 1, 2019; the second indicator was equal to 1 from December 29, 2019, to December 27, 2020 (the end of the study period). For New York, the indicator was equal to 1 from June 14 to December 27, 2020. For Rhode Island, the indicator was equal to 1 from November 3, 2019, to December 27, 2020. For Washington, the indicator was equal to 1 from November 3, 2019, to January 26, 2020. For all the indicators, 0 represented control states and preintervention periods for intervention states.

#### Covariates

The analysis controlled for the percentage of the state population covered by comprehensive smoke-free air laws (bars, restaurants, and workplaces)^29^ and restrictions on sales to persons younger than 21 years (Tobacco 21 laws),^30,31,32^ tobacco control funding as a percentage of Centers for Disease Control and Prevention–recommended funding level,^33,34^ and state cigarette excise taxes.^35^ Because the EVALI outbreak might have affected states differently and therefore may not have been fully captured in time fixed effects, we controlled for EVALI hospitalizations or deaths per state, aggregated from June 2019 to February 2020. The number of EVALI cases was assumed to be 0 before June 2019 and to stay constant after February 2020.^36^ The model also controlled for the cumulative number of COVID-19 cases and deaths from COVID-19 per 100 000 population in each state during each period.^37^ It also controlled for dates of statewide closure owing to the COVID-19 pandemic and reopening dates^38^ using a dichotomous variable that was equal to 1 if a state was closed during a specific period and 0 if a state was not closed as well as the duration of closure in days.

The analysis also controlled for the mean inflation-adjusted after-tax e-cigarette price per standardized unit. This variable was constructed by dividing after-tax total dollar sales by total standardized unit sales for each product and adjusting for inflation and averaging across all products in each state during each period. The analysis also controlled for percentage of menthol cigarette unit sales from IRI^25^ to account for the menthol cigarette ban in Massachusetts in June 2020, population percentages by race and ethnicity and age group,^39^ median annual household income,^40^ and monthly unemployment rates.^41^ All control variables were measured at the state level.

### Statistical Analysis

Difference-in-differences regression models were used to compare the adjusted change over time in e-cigarette sales for each intervention state (before and after flavor restriction) with the adjusted change over time for the control states that did not implement statewide flavored e-cigarette sales restrictions.^42^ An overall linear DID model was estimated for all the intervention states. The dependent variable was log-transformed 4-week total e-cigarette unit sales per capita. The primary independent variables included indicators for each intervention state during the period of flavor restriction, state fixed effects, time fixed effects, and state-specific linear time trends. The model controlled for other state tobacco control policies, state demographic and economic characteristics, and COVID-19 and EVALI measures. The difference in percentage change in sales between each intervention state and control states was obtained using the following formula: [exponential (coefficients) − 1] × 100. To further assess which flavor types were associated with total sales changes, a separate linear DID regression was performed for each flavor type. The dependent variable was the absolute number of units sold per 100 000 population. The estimated coefficients represent the difference in the change in sales between the intervention and control states.

One of the assumptions of the DID model was that sales trends were parallel for the intervention and control states before the implementation of flavor restrictions. We performed a visual check of this assumption by plotting the unadjusted means of dependent variables over time for both groups and plotting the dependent variables estimated from the linear DID model (adjusted means). We also performed a test of the parallel trends assumption by augmenting the DID model with 2 interaction terms that captured the differences in slopes between intervention and control states during the periods before and after implementation of restrictions. The first was an interaction between a linear time trend, an indicator for the intervention states, and an indicator for preimplementation periods. The second was an interaction between a linear time trend, an indicator for the intervention states, and an indicator for postimplementation periods. The parallel trends assumption held if the coefficient of the first interaction term was not statistically significant.

Statistical significance was set at *P* < .05 for all analyses using a 2-sided test; 95% CIs were also calculated. Standard errors were clustered by states, within which errors might be correlated. Analyses were conducted using Stata, version 17 (StataCorp LLC).

A sensitivity analysis was performed considering that residents in intervention states might have purchased e-cigarette products in bordering states after implementation of restrictions in the intervention states, which might have contaminated the control group and affected its validity as a counterfactual. To test this, a DID analysis was conducted excluding bordering states from the control group. In addition, a sensitivity analysis was conducted using nonstandardized unit sales.

## Results

### e-Cigarette Sales Trends

Means and 95% CIs of all covariates are shown in eTable 1 in the Supplement. Before implementation of statewide flavor restrictions, intervention and control states showed similar trends (eFigures 1-4 in the Supplement). Furthermore, the coefficients of the parallel trends test were not statistically significant. Taken together, these findings support the parallel trends assumption.

Overall, sales remained stable until the end of 2017 and then increased through August 2019, when the EVALI outbreak began. Among control states, sales increased by 184.1% from January 2018 to August 2019. Massachusetts experienced a greater increase in sales during this period (269.0%) (Figure), whereas Washington experienced a smaller increase (117.9%). Mint-flavored e-cigarettes had the largest unit sales shares (44%-58%) in all states during 2019 before the peak of the EVALI outbreak.

**Figure.**
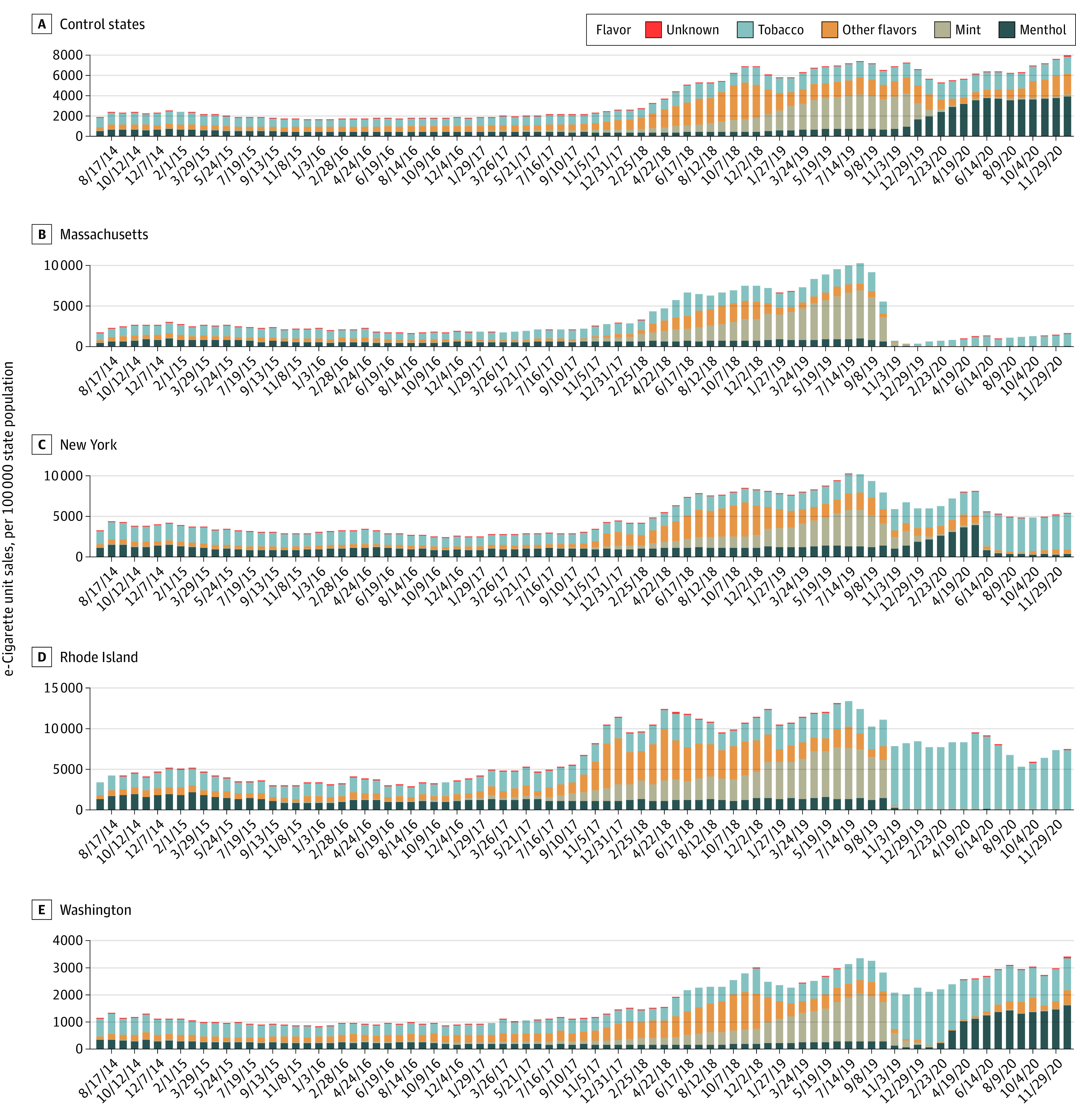
e-Cigarette Unit Sales by Flavor in Intervention States vs Control States From September 2014 to December 2020 Other flavors included fruit; clove or spice; chocolate; alcoholic drink, such as wine, cognac, or other cocktails; candy, desserts, or other sweets; or another flavor. Unknown flavors were excluded from this figure (<0.1%). The control states in each difference-in-differences regression included Alabama, Arizona, Arkansas, California, Colorado, Connecticut, Florida, Georgia, Illinois, Indiana, Iowa, Kentucky, Louisiana, Maine, Maryland, Michigan, Missouri, Nevada, New Hampshire, North Carolina, North Dakota, Ohio, Oklahoma, Oregon, Pennsylvania, South Carolina, South Dakota, Tennessee, Texas, Utah, Vermont, Virginia, West Virginia, Wisconsin, and Wyoming. Each bar represents a 4-week aggregate interval.

Although sales trends were similar from January 2018 to August 2019, trends from August 2019 to December 2020 varied across states. Among control states, mean sales decreased by 28.4% from August 2019 to February 2020 but then increased by 49.9% through December 2020. In December 2020, sales were 7.3% higher than their levels during the start of the EVALI outbreak in August 2019. In contrast to control states, total sales in intervention states decreased consistently from August 2019 to December 2020, by 84.2% in Massachusetts, 46.6% in New York, and 39.6% in Rhode Island. Washington experienced a small increase (1.8%). Furthermore, although menthol-flavored e-cigarettes had the largest unit sales share (49%-59%) in control states, tobacco-flavored e-cigarettes had the largest unit sales share in Massachusetts (>99%), New York (82%-87%), and Rhode Island (>99%). In Washington, where the flavor restriction lasted for only 120 days, tobacco-flavored e-cigarettes had the largest unit sales share (82%-93%) from October 2019 to February 2020, but menthol-flavored e-cigarettes dominated (30%-51%) thereafter.

### Association of Statewide Restrictions With Overall e-Cigarette Sales

After adjustment for COVID-19 and EVALI measures, the results did not meaningfully change, suggesting that time fixed effects and state-specific time fixed effects accounted for most of the secular changes during the study period (Table 1). With use of the results of the model adjusting for COVID-19 and EVALI measures, prohibition of all e-cigarette products in Massachusetts was associated with a 94.38% (95% CI, 93.37%-95.23%) reduction in mean 4-week total e-cigarette unit sales from November 3 to December 1, 2019, compared with the control states. Beginning in December 2019, this policy was narrowed to restrict the sale of non–tobacco-flavored e-cigarettes and was associated with an 88.91% (95% CI, 83.29%-92.64%) reduction in mean 4-week total e-cigarette sales from December 29, 2019, to December 27, 2020, compared with the control states.

**Table 1.** Adjusted Percentage Change in Total e-Cigarette Unit Sales After Implementation of Statewide Flavored e-Cigarette Restrictions in Intervention States Compared With Control States, September 2014 to December 2020^a^

Intervention state (period)	Mean adjusted change per time unit, % (95% CI)^b^		
	Core model	Controlling for COVID-19 pandemic	Controlling for COVID-19 pandemic and EVALI outbreak measures
Massachusetts			
Complete e-cigarette prohibition (October to November 2019)	−94.23 (−94.80 to −93.60)	−94.27 (−94.82 to −93.66)	−94.38 (−95.23 to −93.37)
Flavored e-cigarette prohibition (December 2019 to December 2020)	−88.68 (−91.80 to −84.38)	−88.69 (−92.31 to −83.39)	−88.91 (−92.64 to −83.29)
New York			
Flavored e-cigarette prohibition (May to December 2020)	−35.68 (−42.77 to −27.72)	−33.42 (−40.19 to −25.90)	−30.65 (−36.66 to −24.08)
Rhode Island			
Flavored e-cigarette prohibition (October 2019 to December 2020)	−36.18 (−46.31 to −24.13)	−35.07 (−45.33 to −22.87)	−31.26 (−46.34 to −11.94)
Washington			
Flavored e-cigarette prohibition (October 2019 to January 2020)	−21.94 (−27.58 to −15.86)	−20.57 (−26.17 to −14.55)	−25.01 (−31.05 to −18.43)

Abbreviation: EVALI, e-cigarette or vaping product use–associated lung injury.

^a^ The control states in each difference-in-differences regression included Alabama, Arizona, Arkansas, California, Colorado, Connecticut, Florida, Georgia, Illinois, Indiana, Iowa, Kentucky, Louisiana, Maine, Maryland, Michigan, Missouri, Nevada, New Hampshire, North Carolina, North Dakota, Ohio, Oklahoma, Oregon, Pennsylvania, South Carolina, South Dakota, Tennessee, Texas, Utah, Vermont, Virginia, West Virginia, Wisconsin, and Wyoming.

^b^ The time unit was by 4-week period. Estimates were obtained using difference-in-differences regression. Dependent variables were 4-week log per capita of e-cigarette unit sales in each state (n = 2988). The difference-in-differences models controlled for state fixed effects, 4-week period fixed effects, logarithm of mean real (after tax) price per standardized unit, unit sales share of menthol cigarettes, state characteristics (population by race and ethnicity and age group, median annual household income, and monthly state unemployment rates), and state tobacco control policies (percentage of state population covered by comprehensive smoke-free laws, Tobacco 21 laws, state tobacco control program funding per capita, and state cigarette tax). Reported data are differences in percentage change in sales, calculated as [exponential (coefficients) − 1] × 100.

In New York, prohibition of non–tobacco-flavored e-cigarette sales was associated with a 30.65% (95% CI, 24.08%-36.66%) reduction in mean 4-week total e-cigarette sales from June 14 to December 27, 2020, compared with the control states. In Rhode Island, prohibition of non–tobacco-flavored e-cigarette sales was associated with a 31.26% (95% CI, 11.94%-46.34%) reduction in mean 4-week total e-cigarette sales from November 3, 2019, to December 27, 2020. Washington’s temporary policy from November 3, 2019, to January 26, 2020, was associated with a 25.01% (95% CI, 18.43%-31.05%) reduction in mean 4-week total e-cigarette sales.

### Association of Statewide Restrictions With e-Cigarette Sales by Flavor

Except in Massachusetts, where complete prohibition was associated with a reduction in the sales of all flavors of e-cigarettes, the reductions in total e-cigarette sales were attributable to the reduction in non–tobacco-flavored e-cigarette sales, which was partially compensated by an increase in tobacco-flavored e-cigarette sales (Table 2). The increases in sales of tobacco-flavored e-cigarettes were approximately 40.52%, 43.08%, and 49.17% of the observed total sales decreases in Washington, New York, and Rhode Island, respectively.

**Table 2.** Adjusted Changes in e-Cigarette Unit Sales by Flavor After Implementation of State Flavored e-Cigarette Restrictions in Intervention States Compared With Control States, September 2014 to December 2020^a^

Intervention state (period), flavor	Mean unit change per 100 000 persons per time unit (95% CI)^b^	Unit sales share before intervention, %^c^
Massachusetts (October 2019 to December 2020)		
Tobacco	−1444.38 (−1885.52 to −1003.25)	26.19
Menthol	−1675.70 (−2356.31 to −995.083)	8.57
Mint	−3246.75 (−3816.64 to −2676.86)	57.40
Other^d^	−475.82 (−963.94 to 12.31)	7.84
New York (May to December 2020)		
Tobacco	1953.74 (1790.48 to 2117.00)	37.15
Menthol	−3219.49 (−3608.56 to −2830.43)	48.32
Mint	−62.11 (−479.91 to 355.68)	3.29
Other^d^	−1253.42 (−1755.38 to −751.46)	11.23
Rhode Island (October 2019 to December 2020)		
Tobacco	4488.71 (4050.19 to 4927.24)	28.83
Menthol	−2551.87 (−3574.34 to −1529.40)	12.04
Mint	−4039.51 (−4717.00 to −3362.02)	43.53
Other^d^	−2537.42 (−3051.57 to −2023.27)	15.60
Washington (October 2019 to January 2020)		
Tobacco	765.24 (673.59 to 856.89)	24.00
Menthol	−166.51 (−441.10 to 108.07)	9.73
Mint	−1231.67 (−1600.97 to −862.36)	51.45
Other^d^	−490.46 (−752.15 to −228.77)	14.82

^a^ The control states in each difference-in-differences regression included Alabama, Arizona, Arkansas, California, Colorado, Connecticut, Florida, Georgia, Illinois, Indiana, Iowa, Kentucky, Louisiana, Maine, Maryland, Michigan, Missouri, Nevada, New Hampshire, North Carolina, North Dakota, Ohio, Oklahoma, Oregon, Pennsylvania, South Carolina, South Dakota, Tennessee, Texas, Utah, Vermont, Virginia, West Virginia, Wisconsin, and Wyoming.

^b^ The time unit was by 4-week period. Estimates were obtained using difference-in-differences regression. Dependent variables were 4-week e-cigarette unit sales per 100 000 persons in each state (n = 2988). The difference-in-differences models controlled for e-cigarette or vaping product use–associated lung injury and COVID-19 measures, state fixed effects, 4-week period fixed effects, logarithm of mean real (after tax) price per standardized unit, unit sales share of menthol cigarettes, state characteristics (population by race and ethnicity and age group, median annual household income, and monthly state unemployment rates), and state tobacco control policies (percentage of state population covered by comprehensive smoke-free laws, Tobacco 21 laws, state tobacco control program funding per capita, and state cigarette tax).

^c^ Unit sales share was calculated as sales by each flavor divided by total sales, multiplied by 100.

^d^ Other flavors included fruit; clove or spice; chocolate; alcoholic drink, such as wine, cognac, or other cocktails; candy, desserts, or other sweets; or another flavor.

### Sensitivity Analysis

Exclusion of bordering states from the control group did not change the statistical significance or the direction of the findings (eTable 2 in the Supplement). In addition, in the sensitivity analysis conducted using nonstandardized unit sales, the results were similar.

## Discussion

In this study, statewide restrictions on the sale of e-cigarettes were associated with a reduction in state-level e-cigarette sales in intervention states compared with control states. These results persisted after accounting for other factors, including the EVALI outbreak and the COVID-19 pandemic. Except in Massachusetts, where complete prohibition was associated with a reduction in the sales of all flavors, the reductions in total e-cigarette unit sales were attributable to reductions in non–tobacco-flavored e-cigarette sales. The findings of this study are consistent with previous evaluations of flavored tobacco product restrictions at the substate level in New York City^17^; Lowell, Massachusetts^19,20^; and Minneapolis, St Paul, Duluth, and Falcon Heights in Minnesota.^22,23^

The sales assessed in this study included products purchased by adults and those that could have been directly or indirectly obtained by youths. Given that flavors have been associated with initiation and continued use of e-cigarettes by youths,^3,43^ the decrease in sales of these products would have likely included products obtained directly or indirectly by youths; for example, three-quarters of youths who use JUUL e-cigarettes, the most commonly sold e-cigarette brand in the US, reported purchasing it from a retail store.^44^ Therefore, restrictions on non–tobacco-flavored e-cigarette sales may be an important part of a comprehensive approach to reducing youths’ access to and use of flavored e-cigarettes.

Reductions in total e-cigarette sales were attributable mostly to non–tobacco-flavored (eg, menthol, mint, and other) e-cigarette sales, which were partially compensated by an increase in tobacco-flavored e-cigarette sales in all 4 states. However, the increase in sales of tobacco-flavored e-cigarettes was of lower magnitude than the reduction in sales of non–tobacco-flavored e-cigarettes, leading to an overall decrease in total sales. These findings suggest that not all e-cigarette users who purchased non–tobacco-flavored e-cigarettes switched to purchasing tobacco-flavored e-cigarettes after policy implementation. Subsequent tobacco use behavior of e-cigarette users who did not switch to tobacco flavors remains unclear. Further research of self-reported behaviors is warranted to evaluate the association of flavor restrictions with overall patterns of tobacco product use, including the extent of cessation or substitution behaviors.

The decrease in sales from 2019 to 2020 in intervention states after the implementation of e-cigarette flavor restrictions occurred at the same time as the decrease in current e-cigarette use among US high school (27.5%-19.6%) and middle school (10.5%-4.7%) students.^45^ In addition to state and local actions to restrict flavored e-cigarettes, the decrease in sales also occurred at the same time as the EVALI outbreak, a federal law raising the minimum age of sale for tobacco products to 21 years (December 2019), and the US Food and Drug Administration’s January 2020 actions, and our analysis controlled for these potential confounding factors. Among US adults, current e-cigarette use remained relatively consistent during the assessed period.^46^ Self-reported cigarette smoking trended downward nationally among both adults and youths as e-cigarette use decreased.^46,47^ One study found that the ban on flavored tobacco product sales in San Francisco, California, was associated with increased cigarette smoking among high school students in that city; however, the study did not account for some key measures, including youth e-cigarette use.^48^

### Limitations

This study has limitations. First, sales data did not include online or vape shop sales. Second, sales data did not contain information on purchaser demographics, such as age. Third, ambiguous or concept flavors were back-coded using online searches and might have been subject to misclassification; however, this coding only applied to 5.6% of total sales. Fourth, this analysis did not account for state differences in policy implementation and enforcement. Fifth, factors other than the flavor-related policies could have been associated with e-cigarette sales.^49^ However, steps were taken to account for such factors, including controlling for co-occurring factors, such as the EVALI outbreak, the COVID-19 pandemic, and other population-based policies.

## Conclusions

In this study, statewide non–tobacco-flavored e-cigarette restrictions were associated with a reduction in total e-cigarette sales in intervention states compared with control states. The reduction in total sales was attributable to a decrease in non–tobacco-flavored e-cigarette sales, suggesting that not all e-cigarette users who purchased non–tobacco-flavored e-cigarettes switched to purchasing tobacco-flavored e-cigarettes after policy implementation. Given the appeal of flavors for prompting use of tobacco products among youths,^3,4,5,6^ we suggest that comprehensive policies that prohibit the sale of all non–tobacco-flavored e-cigarettes to prevent and reduce youth access to and use of these products should be implemented. Noncomprehensive policies, such as those that exempt certain flavors, may diminish effects.^50^

## Notes

Supplement. **eFigure 1.** Graphical Diagnostics for Parallel Trends in Massachusetts **eFigure 2.** Graphical Diagnostics for Parallel Trends in New York **eFigure 3.** Graphical Diagnostics for Parallel Trends in Rhode Island **eFigure 4.** Graphical Diagnostics for Parallel Trends in Washington **eTable 1.** Means and 95% Confidence Intervals of Covariates Included in the Analysis **eTable 2.** Adjusted Percentage Change in Total e-Cigarette Unit Sales Following Implementation of State Flavored e-Cigarette Restrictions, Compared to Non-Bordering Control States-USA, September 2014-December 2020
