# Supplementary material for: Evaluation of Statewide Restrictions on Flavored e-Cigarette Sales in the US From 2014 to 2020
Source: JAMA Netw Open. 2022 Feb 10;5(2):e2147813. doi: 10.1001/jamanetworkopen.2021.47813 (PMC8832173; doi:10.1001/jamanetworkopen.2021.47813)
Supplement: Supplement. — eFigure 1. Graphical Diagnostics for Parallel Trends in Massachusetts eFigure 2. Graphical Diagnostics for Parallel Trends in New York eFigure 3. Graphical Diagnostics for Parallel Trends in Rhode Island eFigure 4. Graphical Diagnostics for Parallel Trends in Washington eTable 1. Means and 95% Confidence Intervals of Covariates Included in the Analysis eTable 2. Adjusted Percentage Change in Total e-Cigarette Unit Sales Following Implementation of State Flavored e-Cigarette Restrictions, Compared to Non-Bordering Control States-USA, September 2014-December 2020 [file jamanetwopen-e2147813-s001.pdf]

Supplementary Online Content

Ali FRM, Vallone D, Seaman EL, et al. Evaluation of statewide restrictions on flavored e-cigarette sales in the US from 2014 to 2020. *JAMA Netw Open*. 2022;5(2):e2147813. doi:10.1001/jamanetworkopen.2021.47813

eFigure 1. Graphical Diagnostics for Parallel Trends in Massachusetts
eFigure 2. Graphical Diagnostics for Parallel Trends in New York
eFigure 3. Graphical Diagnostics for Parallel Trends in Rhode Island
eFigure 4. Graphical Diagnostics for Parallel Trends in Washington
eTable 1. Means and 95% Confidence Intervals of Covariates Included in the Analysis
eTable 2. Adjusted Percentage Change in Total e-Cigarette Unit Sales Following Implementation of State Flavored e-Cigarette Restrictions, Compared to Non-Bordering Control States-USA, September 2014-December 2020

This supplementary material has been provided by the authors to give readers additional information about their work.

© 2022 Ali FRM et al. *JAMA Network Open*.

Figure 1. Graphical diagnostics for parallel trends in Massachusetts

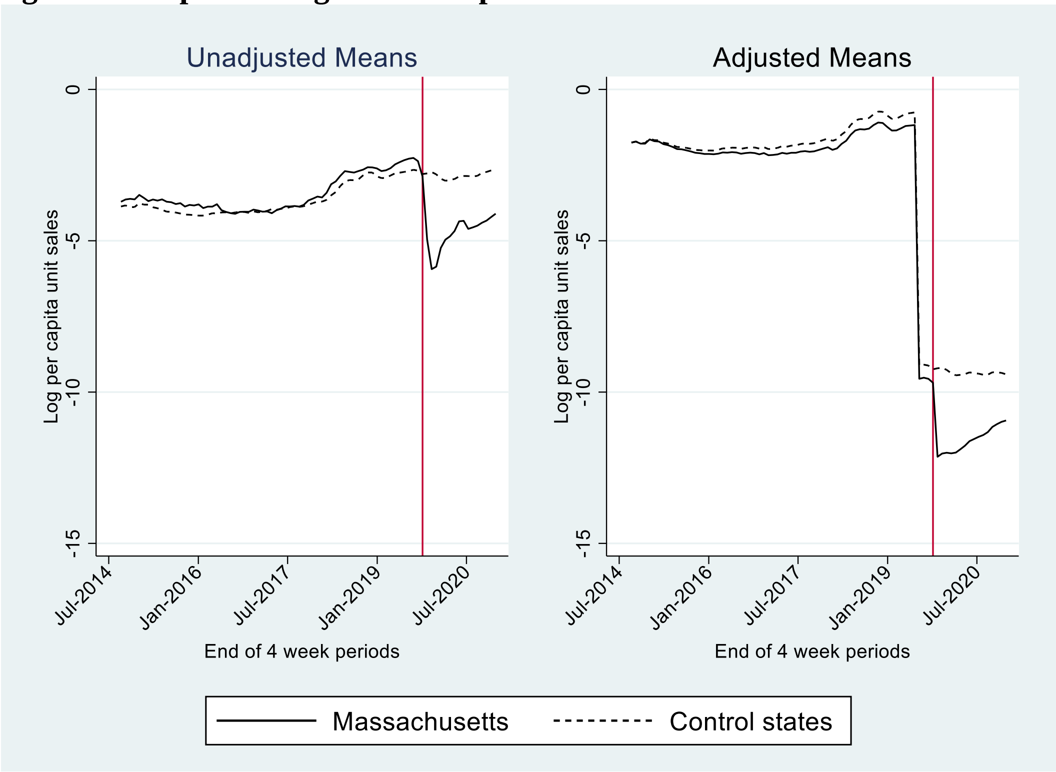

Figure 2. Graphical diagnostics for parallel trends in New York

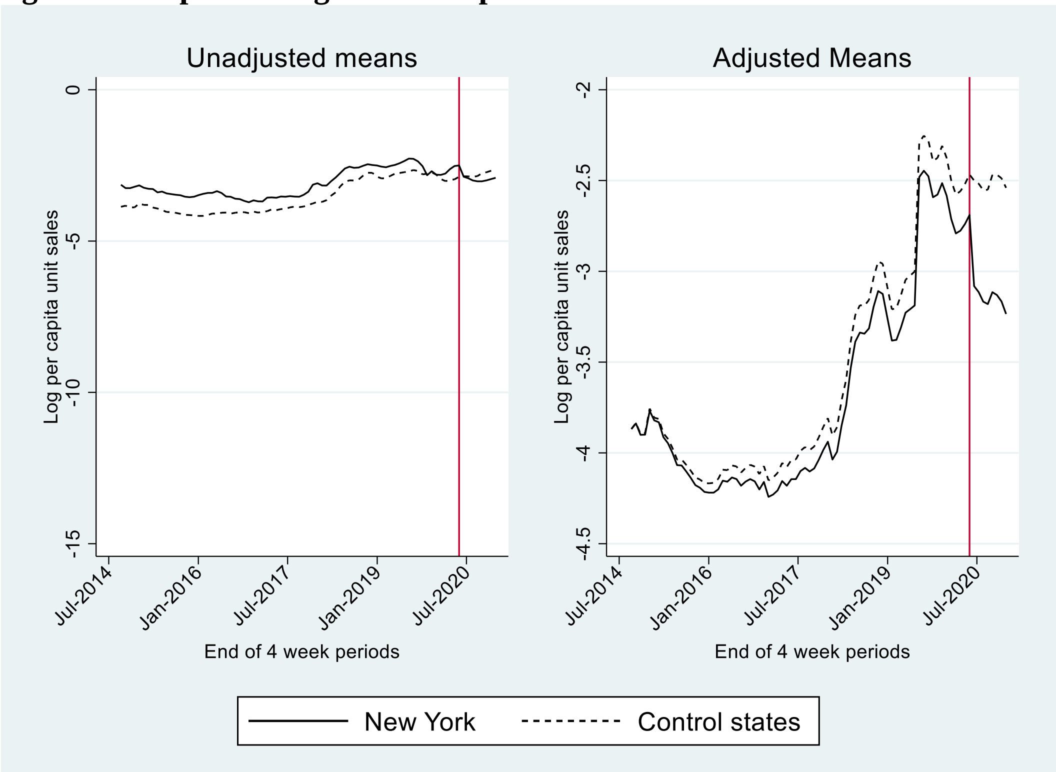

© 2022 Ali FRM et al. *JAMA Network Open*.

Figure 3. Graphical diagnostics for parallel trends in Rhode Island

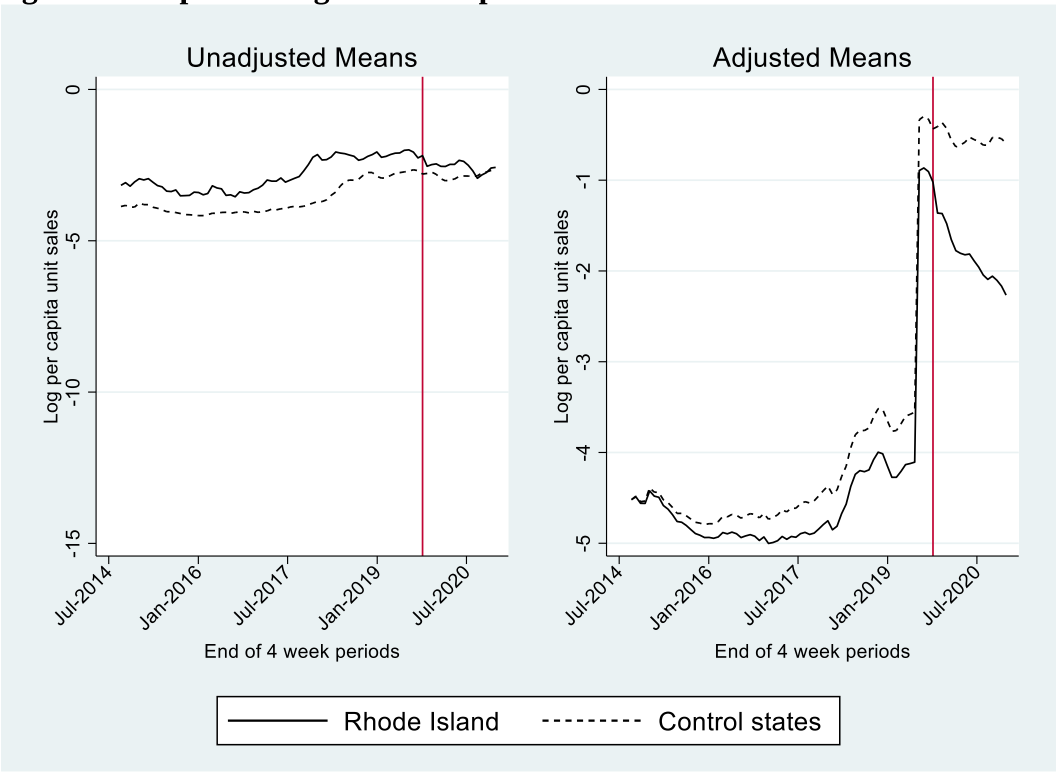

Figure 4. Graphical diagnostics for parallel trends in Washington

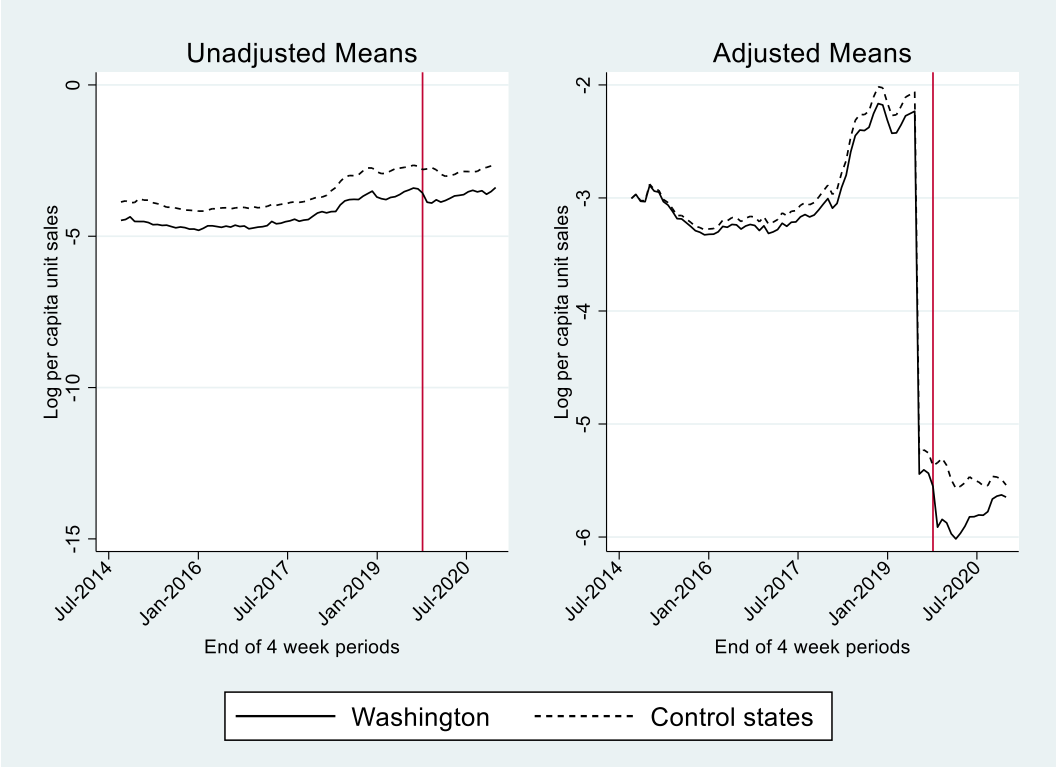

© 2022 Ali FRM et al. *JAMA Network Open*.

Table 1. Means and 95% Confidence Intervals of Covariates included in the Analysis

Covariates	Massachusetts	New York	Rhode Island	Washington	Control states
Age groups					
under 18	19.98 (19.63 to 20.6)	20.97 (20.71 to 21.41)	19.59 (19.3 to 20.11)	22.17 (21.84 to 22.71)	22.52 (18.27 to 30.77)
Age 18-24	10.17 (10.02 to 10.38)	9.33 (9.06 to 9.92)	10.64 (10.34 to 11.16)	8.89 (8.65 to 9.45)	9.49 (7.95 to 12.33)
Age 25-44	26.45 (26.21 to 26.74)	27.1 (27.01 to 27.23)	25.45 (25.02 to 25.82)	28.34 (27.49 to 28.89)	25.86 (22.73 to 29.72)
Age 45-64	27.16 (26.65 to 27.69)	26.44 (26.06 to 26.78)	27.41 (26.88 to 27.93)	25.44 (24.73 to 26.37)	25.95 (19.7 to 30.63)
Age 65 or higher	16.24 (15.09 to 16.97)	16.16 (14.88 to 16.94)	16.91 (15.77 to 17.66)	15.16 (13.97 to 15.89)	16.19 (10.02 to 21.22)
Race groups					
None Hispanic whites	72.14 (71.06 to 74.18)	55.79 (55.29 to 56.83)	72.48 (71.36 to 74.45)	68.61 (67.51 to 70.48)	70.79 (36.5 to 93.76)
None Hispanic blacks	7.16 (6.83 to 7.34)	14.49 (14.46 to 14.56)	5.96 (5.69 to 6.13)	3.88 (3.7 to 4)	11.27 (1.02 to 32.29)
Hispanics	11.87 (10.89 to 12.4)	19.03 (18.53 to 19.28)	15.51 (14.13 to 16.3)	12.69 (12.06 to 13.02)	11.26 (1.45 to 39.75)
Others	8.82 (8.11 to 9.2)	10.68 (10.08 to 10.97)	6.05 (5.73 to 6.21)	14.83 (13.76 to 15.47)	6.67 (2.52 to 18.46)
Monthly unemployment rate	4.12 (1.2 to 9.5)	4.91 (2.5 to 9.4)	4.73 (0.1 to 7.9)	5.04 (0 to 9.9)	4.41 (0 to 11.8)
Median annual household income	81473.98 (68264 to 87909)	66947.81 (58707 to 71855)	66145 (60107 to 70151)	77302 (63850 to 82454)	63670.16 (42754 to 95572)
Cigarette excise tax ($)	3.51 (3.51 to 3.51)	4.35 (4.35 to 4.35)	3.98 (3.5 to 4.25)	3.03 (3.03 to 3.03)	1.91 (0.17 to 52.6)
%Population covered by SFA[a]	100 (100 to 100)	100 (100 to 100)	100 (100 to 100)	100 (100 to 100)	47.7 (0 to 100)
Tobacco 21[b]					
0	0.82 (0 to 1)	0.81 (0 to 1)	0.82 (0 to 1)	0.82 (0 to 1)	0.79 (0 to 1)
1	0.18 (0 to 1)	0.19 (0 to 1)	0.18 (0 to 1)	0.18 (0 to 1)	0.21 (0 to 1)
%TC CDC funding[c]	6.02 (5.6 to 6.9)	19.46 (19.4 to 19.6)	3.01 (2.9 to 3.1)	2.53 (1 to 3.6)	20.25 (0 to 102)
Log e-cigarette price[d]	2.87 (2.37 to 3.49)	2.77 (2.35 to 3.04)	2.85 (2.36 to 3.18)	2.74 (2.34 to 3.05)	2.76 (2.21 to 3.61)
%Menthol cigarette unit sales	23.64 (0.15 to 29.85)	35.4 (33.4 to 36.82)	40.93 (38.62 to 45.59)	22.92 (21.6 to 24.66)	29.72 (14.61 to 53.52)
Covid cases per 100,000 people	258.21 (0 to 5057.67)	144.61 (0 to 2646.72)	326.05 (0 to 7746.75)	132.48 (0 to 3118.01)	243.89 (0 to 12014.51)
Covid death per 100,000 people	14.61 (0 to 174.97)	5.38 (0 to 62.61)	11.25 (0 to 160.85)	2.91 (0 to 41.81)	4.86 (0 to 166.65)
Covid closure dummy					
0	0.96 (0 to 1)	0.95 (0 to 1)	0.98 (0 to 1)	0.95 (0 to 1)	0.97 (0 to 1)
1	0.04 (0 to 1)	0.05 (0 to 1)	0.02 (0 to 1)	0.05 (0 to 1)	0.03 (0 to 1)
Duration of Covid closure (days)	2.75 (0 to 76)	3.81 (0 to 79)	1.01 (0 to 42)	4.92 (0 to 102)	1.64 (0 to 116)
EVALI cases					

© 2022 Ali FRM et al. *JAMA Network Open*.

0 cases	0.76 (0 to 1)	0.76 (0 to 1)	0.76 (0 to 1)	0.76 (0 to 1)	0.76 (0 to 1)
1-9 cases	0.24 (0 to 1)	0.24 (0 to 1)	0.24 (0 to 1)	0.24 (0 to 1)	0.05 (0 to 1)
10-49 cases	0	0	0	0	0.08 (0 to 1)
50-99 cases	0	0	0	0	0.06 (0 to 1)
100-149 cases	0	0	0	0	0.03 (0 to 1)
150-199 cases	0	0	0	0	0.01 (0 to 1)
200-249 cases	0	0	0	0	0.01 (0 to 1)

[a] This variable represents the percentage of state population covered by comprehensive smoke-free air (SFA) laws (bars, restaurants, and workplaces)
[b] This variable represents state laws restricting tobacco product sales to persons under 21
[c] This variable represents tobacco control funding as a percentage of CDC recommended funding level.
[d] This variable represents mean inflation-adjusted after-tax e-cigarette price per standardized unit. This variable was constructed by dividing after-tax total dollar sales by total standardized unit sales for each UPC product, and adjusting for inflation and averaging across all UPC products in each state in each period.

Table 2. Adjusted Percentage Change in Total E-cigarette Unit Sales Following Implementation of State Flavored E-cigarette Restrictions, Compared to Non-Bordering Control States-USA, September 2014-December 2020

	**Excluding bordering States from the Control States[c]**
**Intervention State**	**Adjusted % Change[a] Per Time Unit[b] (95% CI)**
**Massachusetts**	
Complete E-cigarette Prohibition (Oct 2019-Nov 2019)	-94.16 (-95.02 to -93.15)
Flavored E-cigarette Prohibition (Dec 2019-Dec 2020)	-89.12 (-92.17 to -84.86)

© 2022 Ali FRM et al. *JAMA Network Open*.

**New York**	
Flavored E-cigarette Prohibition (May 2020-Dec 2020)	-26.80 (-33.79 to -19.07)
**Rhode Island**	
Flavored E-cigarette Prohibition (Oct 2019-Dec 2020)	-35.64 (-51.88 to -13.91)
**Washington**	
Flavored E-cigarette Prohibition (Oct 2019-Jan 2020)	-28.61 (-34.80 to -21.84)

Note: CI = confidence interval.
Estimates were obtained using separate linear difference-in-differences (DID) regression models, controlling for Covid and EVALI measures. Dependent variables were 4-week log per capita of e-cigarette unit sales in each state. Each DID model controlled for state fixed effects, 4-week period fixed effects, logarithm of mean real (after tax) price per standardized unit, unit sales share of menthol cigarettes, state characteristics (population by race/ethnicity and age group, median annual household income, and monthly state unemployment rates), state tobacco control policies (percentage of state population covered by comprehensive smoke-free laws, Tobacco 21 laws, state tobacco control program funding per capita, and state cigarette tax). Reported data 8 are percentage change in sales, calculated as [Exponential (Coefficients) − 1] ×100.
[b]Time unit is by 4-week period.
[c] The control states in each regression included the control states in the main model (Alabama, Arizona, Arkansas, California, Colorado, Connecticut, Florida, Georgia, Illinois, Indiana, Iowa, Kentucky, Louisiana, Maine, Maryland, Michigan, Missouri, Nevada, New Hampshire, North Carolina, North Dakota, Ohio, Oklahoma, Oregon, Pennsylvania, South Carolina, South Dakota, Tennessee, Texas, Utah, Vermont, Virginia, West Virginia, Wisconsin, Wyoming), excluding the bordering states; for Massachusetts, it excluded Connecticut, Pennsylvania, and Vermont; for New York, it excluded Connecticut, Pennsylvania, and Vermont; for Rhode Island, it excluded Connecticut; and for Washington, it excluded Oregon.

© 2022 Ali FRM et al. *JAMA Network Open*.
